# The risk of clopidogrel resistance is associated with ABCB1 polymorphisms but not promoter methylation in a Chinese Han population

**DOI:** 10.1371/journal.pone.0174511

**Published:** 2017-03-30

**Authors:** Jia Su, Qinglin Yu, Hao Zhu, Xiaojing Li, Hanbin Cui, Weiping Du, Lindan Ji, Maoqing Tong, Yibo Zheng, Hongyu Xu, Jianjiang Zhang, Yunyun Zhu, Yezi Xia, Ting Liu, Qi Yao, Jun Yang, Xiaomin Chen, Jingbo Yu

**Affiliations:** 1 Department of Gerontology, Ningbo No.1 Hospital, Ningbo, Zhejiang Province, People's Republic of China; 2 Department of Traditional Chinese Internal Medicine, Ningbo No.1 Hospital, Ningbo, Zhejiang Province, People's Republic of China; 3 Department of Anaesthesia, Ningbo No.1 Hospital, Ningbo, Zhejiang Province, People's Republic of China; 4 Department of Cardiology, Ningbo No.1 Hospital, Ningbo, Zhejiang Province, People's Republic of China; 5 Department of Biochemistry, School of Medicine, Ningbo University, Ningbo, Zhejiang Province, People's Republic of China; 6 The Key Laboratory of Molecular Medicine, Ningbo No. 1 Hospital, School of Medicine, Ningbo University, Ningbo, Zhejiang, China; Universitatsklinikum Freiburg, GERMANY

## Abstract

The goal of our study was to investigate the contribution of *ABCB1* expression to the risk of clopidogrel resistance (CR). Platelets functions were measured using the Verify-Now P2Y12 assay. Applying Polymerase Chain Reaction–Restriction Fragment Length Polymorphism (PCR-RFLP), the single-nucleotide polymorphisms (SNPs) was tested. Using bisulphite pyrosequencing assay, we investigated the association of the *ABCB1* DNA methylation levels and CR. It was shown that female, hypertension, and lower albumin levels increased the risk of CR (P<0.05). If patients did not have hypoproteinaemia or had hypertension, the SNP in rs1045642 was associated with CR (CC vs. TT: albumin ≥35, *P* = 0.042; hypertension, *P* = 0.045; C vs. T: albumin ≥35, *P* = 0.033; hypertension, *P* = 0.040). Additionally, the platelet inhibition of the CT+TT genotype in rs1128503 was larger than that of the CC genotype (P = 0.021). Multivariate logistic regression analysis showed that male, higher albumin and hsCRP decreased the risk of CR, and the stent size maybe positively correlated with CR. The SNP in rs1045642 was related to all-cause mortality (*P* = 0.024). We did not find any relationship between the methylation levels of the *ABCB1* promoter and CR. In conclusions, our study indicated that ABCB1 polymorphisms might be useful in further evaluating the pathogenesis of CR.

## Introduction

At sites of vascular injury due to atherosclerotic plaque rupture or erosion, platelets mediate not only haemostasis but also pathologic thrombosis[1]. Thrombus generation (due to platelet activation and aggregation) is the main process involved in atherosclerotic vascular disease, particularly coronary artery disease (CAD)[2]. Therefore, antiplatelet therapy has been the cornerstone therapy in patients with coronary artery disease, especially in those undergoing percutaneous coronary intervention (PCI)[3].

Through inhibiting the purinergic ADP receptor P2Y12, clopidogrel reduces adenosine diphosphate-induced platelet aggregation and decreases the risk of cardiovascular events in CAD patients[4]. However, a large number of patients continue to suffer recurrent ischaemic events[5], and this clinical phenomenon has been correlated with lesser degrees of platelet inhibition[6]. This failure of the antiplatelet drug to inhibit its target of action is called clopidogrel non-responsiveness or clopidogrel resistance[7]. Recently, both prasugrel and ticagrelor, which are novel and stronger antiplatelet agents, were shown to exert more consistent, rapid and effective P2Y12 receptor inhibition in patients with acute coronary syndrome (ACS)[8]. Nevertheless, high incidence of major bleeding in some patients receiving prasugrel was noted[9], and ticagrelor was associated with an 11% increase in combined major and minor PLATO bleeding rates after careful analysis of bleeding events[10]. And cases of inadequate platelet inhibition of prasugrel had been occasionally reported however the incidence of this is less than clopidogrel resistance and this is in keeping with prasugrel being a prodrug[11]. Therefore, clopidogrel remains to be one of the most extensively prescribed antiplatelet drugs in CAD patients, and research focused on the individual susceptibility to clopidogrel is of vital significance.

Several clinical and demographic factors may influence the antiplatelet efficacy of clopidogrel, such as drug-drug interactions (such as Proton pump inhibitors[12]), renal dysfunction, diabetes mellitus (DM), diet, smoking, age, reduced left ventricular function, inflammation and the presence of an ACS[13]. However, genetic factors, specifically the expression of the ABCB1 gene, may significantly influence clopidogrel’s response[14].

Clopidogrel is an oral, second-generation thienopyridine irreversible inhibitor of the P2Y12 receptor. It undergoes rapid absorption by the duodenum and is metabolized by hepatic cytochrome P450 enzymes. About 15% of clopidogrel’s prodrug is converted into a biologically active thiol metabolite, which, in circulation, irreversibly combines to and inactivates the P2Y12 receptor on the surfaces of platelets, resulting in the inhibition of ADP-induced platelet activation and aggregation[15]. In the above transformation, specific genetic variants are responsible for clopidogrel’s transport (ATP-binding cassette subfamily B member 1 [ABCB1]), metabolism (CYP enzymes, paraoxonase-1) and action (P2Y12)[16].

The *ABCB1* gene, which is also called *MDR1* or *TAP1*, encodes the intestinal efflux transporter pump P-glycoprotein and modulates the absorption of clopidogrel[17]. The effect of different levels of ABCB1 expression is unclear. Simon *et al*.[18] first analysed the influence of the ABCB1 C3435T (rs1045642) polymorphism on clinical outcomes in persons treating with clopidogrel and found that homozygous patients had a higher risk of cardiovascular events. One recent study was found that exposition to clopidogrel, measured by AUC0-t of the drug, was significantly lower in TT homozygotes comparing to CC and CT genotypes (ABCB1 3435C>T), which showed that the presence of 3435C>T allele had an impact on clopidogrel pharmacokinetics[19]. However, these findings could not be confirmed in several subsequent studies. Additionally, the effect of the rs1128503 polymorphism and the DNA methylation of selected CpG islands in the ABCB1 gene on clopidogrel’s response are poorly understood. Thus, in the present study, we attempted to assess whether the rs1045642 and rs1128503 polymorphisms and DNA methylation in the ABCB1 promoter are involved in clopidogrel resistance in Chinese CAD patients treated with clopidogrel.

## Methods

### Study population

From 2010 to 2015, a total of 180 patients with acute coronary syndromes (ACS) were enrolled in present study in the Ningbo No. 1 Hospital. All of them were Han Chinese from Ningbo city in Eastern China. The inclusion criteria were as follows: (1) According to the most recent guidelines (ACC/AHA guidelines), all individuals were undergoing PCI through the radial route via a drug-eluting stent. The patients we tested the platelet function were mostly existed multi-vessel or left main vessel disease of the coronary artery. (2) The patients received clopidogrel (300 mg) and aspirin (300 mg) before PCI and were administered clopidogrel 75 mg and aspirin 100 mg daily. (3) The patients’ age was required to be ≥18 years. The exclusion criteria were as follows: (1) the therapy of concomitant glycoprotein IIb/IIIa inhibitor or warfarin, (2) hepatic or renal insufficiency, (3) history of active bleeding diathesis, (4) hepatic and renal insufficiency, and (5) total platelet count < 150 000 μl^-1^ or > 500 000 μl^-1^. All patients provided written informed consent before participation in the research. The study protocol was reviewed and approved by the Ethics Committee of Ningbo No1. Hospital and conformed to the guide lines of the Declaration of Helsinki.

### Collection of blood samples and clinical data

Blood samples were obtained overnight via venipuncture in the antecubital area. Serologic markers, such as the concentrations of TC, TG, LDL, GLU, HbA1c, BUN, and CREA, were detected. All the measures applied the standard procedures, and data were collected.

### Platelet function measurements

With the administration of clopidogrel for 3 to 5 days, the platelet reactivity may have been stable or not significantly changed in AMI patients undergoing PCI[20]. We detected the platelet reactivity one month after PCI. By the double-syringe technique, blood samples were gathered and the first 5 ml was discarded to avert unprompted platelet activation. The platelet function measurements were analysed by the VerifyNow *P2Y12* assay (Accumetrics Inc., San Diego, California), which was developed to assess the response to *P2Y12* antagonists[21]. The VerifyNow *P2Y12* assay reported *P2Y12* reaction units (PRU), and a PRU more than 240 reaction units suggested the existence of clopidogrel resistance[22].

### Genomic DNA extraction, genotyping, and methylation assay

Human genomic DNA was extracted from 3 ml peripheral blood by QIAamp DNA BloodMini Kit (Qiagen). Samples were stored at -100°C until use. PCR primers were planned through PyroMark Assay Design software. The sequences of primers used in the SNP genotyping and DNA Methylation Assay are described in ***Table 1***.

**Table 1:** The primers for SNP genotyping and DNA methylation assay.

	Group	DNA sequence
SNP genotyping	rs1045642-F	5-'GTGTGCTGGTCCTGAAGTTG-3'
	rs1045642-R	5'- TGGAGCCTCAAGCCTATAGC -3'
	rs1128503-F	5-'GTTCACTTCAGTTACCCATCTCG-3'
	rs1128503-R	5'- CGTGGTGGCAAACAATACAGG -3'
DNA Methylation Assay	ABCB1-F1	GGATATGGAAGTTAAGATTTTAGAGATA
	ABCB1-R1	ATTTCAAATATCCCATTACCACATATAAC
	ABCB1-S1	ATGATTAATGAGGTAGAAAAAAG

Polymerase chain reaction (PCR) amplification (BIO-RAD C1000touch Thermal Cycler PCR) was performed as follows: 50 μl reaction volume containing 1 μl template DNA, 1.5 μl 10 mM dNTP, 5 μl Taq Buffer, 1.0 μl25 mM MgCl_2_, 1.5 μl upstream and downstream primers, 1 μl platinum Taq polymerase 1 U and 37.5 μl water. PCR cycling conditions consisted of 2 cycles of 94°C denaturation for 2 min, 30 cycles of 94°C denaturation for 30 sec, 55°C annealing for 45 sec, and 68°C extension for 3 min, and one cycle of 68°C repair extension for 10 min. PCR products were purified (SK1141; kit Sangon Biotech), measured (3500XL sequence analyser; ABI), and sequenced.

The bisulphite pyrosequencing technology was applied to evaluate the quantitative DNA methylation of 2 CpG dinucleotides on the fragment of ABCB1 gene promoter[23]. This process was also combined with sodium bisulphite DNA conversion chemistry (EpiTech Bisulphite Kits; Qiagen), polymerase chain reaction amplification (Pyromark PCR Kit; Qiagen) and sequencing (Pyromark Gold Q24 Reagents; Qiagen) of the target fragment[24].

### Statistical analysis

Quantitative data are described as the mean ± standard deviation for categorical variables or the median with interquartile range (IQR) for continuous variables. We carried out a suite of statistical analyses to investigate the association among genetic variables, various confounding factors and clopidogrel resistance. Categorical variables were analysed with either Pearson’s chi-square test or Fisher’s exact test when appropriate. Continuous variance with normality and homogeneity were applied to compare the mean values from *t*-tests. Non-parametric continuous variance was performed using the Wilcoxon rank-sum test. Logistic regression was used to test the interaction of ABCB1 SNP and confounding variables. All the statistical analyses were conducted by PASW Statistics 18.0 software (SPSS, Inc., Somers, NY, USA). A value of P less than 0.05 was considered to indicate a statistically significant difference.

## Results

### Study population

From May 2010 to October 2015, 180 CAD patients who met all requirements were recruited for the present study. UsingVerifyNowP2Y12 assay, 81 patients whose PRU was greater than 240 were defined as having a poor clopidogrel response or clopidogrel resistance. The demographic and clinical characteristics of the cases and controls are summarized in ***Table 2***. The clinical information was well matched except for albumin and the ratios of gender and hypertension. Patients with poor clopidogrel response were more likely to be female (cases versus controls: 32.1% versus 15.15%, *P* = 0.007) and have hypertension (cases versus controls: 75.31% versus 58.59%, *P* = 0.018) and lower albumin levels (cases versus controls:38.11 ± 4.38 versus 39.83 ± 5.64, *P* = 0.026). These data indicated that female sex, hypertension and lower albumin levels might increase the risk of clopidogrel resistance.

**Table 2:** Comparison between CR and non-CR characteristics.

	CR (81)	Non-CR (99)	*P* value
Hypertension, n(%)	61 (75.31)	58 (58.59)	0.018
Diabetes mellitus, n(%)	16 (19.75))	22 (22.22)	0.686
Hyperlipidemia, n(%)	32 (39.51)	44 (44.44)	0.505
Current smoking, n(%)	31 (38.27)	42 (42.42)	0.572
Alcohol abuse, n(%)	17 (20.99)	24 (24.24)	0.604
Gender (male), n(%)	55 (67.90)	84 (84.85)	0.007
Age, y	65.69 ±11.85	63.01 ±12.06	0.136
BMI, kg/m^2^	23.29 ±4.58	24.41 ±10.15	0.363
TC, mg/dL	4.36 ±3.72	4.77 ±1.48	0.273
TG, mg/dL	1.67 ±1.16	1.70 ±1.33	0.880
HDL, mg/dL	0.96 ±0.27	1.00 ±0.28	0.370
LDL, mg/dL	2.77 ±0.98	2.86 ±1.02	0.533
GLU, mmol/L	6.09 ±2.10	5.88 ±2.42	0.535
HbA1c, %	6.17 ±1.08	6.49 ±3.36	0.420
ALT, μmol/L	36.67 ±31.47	37.36 ±29.52	0.879
AST, μmol/L	109.75 ±162.84	113.29 ±167.46	0.887
TBIL, μmol/L	15.29 ±8.51	14.77 ±8.03	0.676
Album, g/L	38.11 ±4.38	39.83 ±5.64	0.026
BUN, mmol/L	5.98 ±2.27	5.46 ±1.97	0.101
CREA, mmol/L	75.37 ±24.93	72.71 ±22.44	0.453
UA, μmol/L	350.88 ±129.78	322.37 ±80.42	0.073
hsCRP, mg/L	12.24 ±19.81	14.07 ±26.21	0.604
PLT, *10^9^/L	194.30 ±58.05	201.43 ±73.93	0.480
MPV, fL	8.38 ±1.32	8.09 ±0.93	0.088
PCT, %	0.16 ±0.04	0.16 ±0.05	0.953
PDW, %	16.23 ±1.19	16.33 ±0.58	0.488
LEVF, %	59.40 ±9.40	61.28 ±7.54	0.137
Stent, n	1.57 ±0.95	1.31 ±0.79	0.051

### Clopidogrel resistance and thers1045642 and rs1128503 polymorphisms

It was well known that the variance of rs1045642 and rs1128503is common in Chinese populations. We searched the genetic information of CHB on the HAP-MAP and found that the frequency of the C allele in ABCB1 3435C>T (rs1045642) and the C allele in ABCB1 1236C>T (rs1128503) in Chinese populations are 0.613 and 0.293. We calculated the frequency of these two SNP in our study and ensured that each of the target polymorphisms was in Hardy–Weinberg equilibrium (***Table 3***).

**Table 3:** Hardy-Weinberg equilibrium test of rs1045642 and rs1128503.

	ABCB1 3435C>T (rs1045642)					ABCB1 1236C>T (rs1128503)				
	CC	CT	TT	Chi-squre	HWE	CC	CT	TT	Chi-squre	HWE
N	76	79	25	0.374	0.54	39	92	59	0.082	0.76

Through PCR-HPCE, we explored the association of clopidogrel resistance and the rs1045642 and rs1128503 polymorphisms. As shown in ***Table 4***, our results showed that the variation of rs1045642 and rs1128503 was not significantly associated with clopidogrel resistance (*P*_*rs1045642*_ = 0.288; *P*_*rs1128503*_ = 0.644).

**Table 4:** Comparison of the SNP in rs1045642 and rs1128503 between cases and controls.

	ABCB1 3435C>T (rs1045642)				ABCB1 1236C>T (rs1128503)			
	CC	CT	TT	*P value*	CC	CT	TT	*P value*
CR	38	35	8	0.228	13	40	28	0.644
NCR	38	44	17		16	52	31	

Additionally, we determined the relationship by the comparison of the different SNP modes (homozygotes and dominant, recessive and heterozygous models), and the results were insignificant (***Table 5***).

**Table 5:** Comparison of the different SNP modes in two loci between cases and controls.

	CC+TT vs. TT		*P value*	CC vs. CT+TT		*P value*	CC vs. TT		*P value*	C vs. T		*P value*
ABCB13435C>T (rs1045642)												
CR	73	8	0.159	38	43	0.249	38	8	0.117	111	51	0.012
NCR	82	17		38	61		38	17		110	88	
ABCB11236C>T (rs1128503)												
CR	53	28	0.644	13	68	0.984	13	28	0.816	66	96	0.747
NCR	68	31		16	85		16	31		84	114	

### Subgroup analysis

Due to the differences in the baseline characteristics between the CR and NCR patients, we carried out subgroup analysis according to the ratios of males, hypertension and albumin. Overall, there was no significant difference for any subgroup (***Table 6***).

**Table 6:** Subgroup analysis for SNP in rs1045642 and rs1128503 between cases and controls.

		cases			controls			
		CC	CT	TT	CC	CT	TT	*P value*
*ABCB1*3435C>T (rs1045642)								
Hypertension	Yes	33	26	5	20	27	11	0.075
	No	8	9	3	18	17	16	0.357
Gender	Male	24	26	5	34	36	14	0.445
	Female	14	9	3	4	8	3	0.238
Albumin	≥35	35	50	5	34	39	15	0.123
	<35	3	5	3	4	5	2	0.842
*ABCB1*1236C>T (rs1128503)								
Hypertension	Yes	9	29	23	8	31	19	0.806
	No	4	11	5	8	21	12	0.938
Gender	Male	8	27	20	16	43	25	0.684
	Female	5	13	8	0	9	6	0.192
Albumin	≥35	12	32	26	12	46	29	0.655
	<35	1	8	2	4	6	2	0.359

However, we tested the relationship between platelet activity and the four SNP modes, producing new significance values. For rs1045642, in patients with hypertension or a value of albumin greater than 35, patients with the CC genotype had a higher risk of CR than those with the TT genotype (albumin≥35, P = 0.042; hypertension P = 0.045). At the same time, patients with the C allele were more likely to have an increased risk of CR than those with the T allele (albumin≥35 P = 0.033; hypertension P = 0.040)(***Table 7)***.

**Table 7:** Subgroup analysis for the different SNP modes in rs1045642 between cases and controls.

	CC+TT vs. TT		*P value*	CC vs. CT+TT		*P value*	CC vs. TT		*P value*	C vs. T		*P value*
Albumin≥35												
CR	65	5	0.063	35	35	0.153	35	5	0.042	100	40	0.033
NCR	75	15		34	54		34	15		103	69	
Hypertension												
CR	56	5	0.085	30	31	0.104	30	5	0.045	86	36	0.040
NCR	47	11		20	38		20	11		67	49	

### Different SNP modes and the index of platelet function

The Verify-Now assay measures the agonist-induced activation of platelets and their binding to fibrinogen-coated polystyrene beads. The assay uses ADP as an agonist and PGE1 as an antagonist, and the results are reported as P2Y12 reaction units (PRU)[25].With this test, we obtained the value of PRU, the baseline of platelet activity, and the inhibition value. The inhibition value is equivalent to (baseline-PRU)/baseline. We compared the relationship between the SNP and PRU, as well as inhibition, and did not find any significant results (***Table 8***).

**Table 8:** Comparison of platelet function values and the SNP at the two loci.

		PRU	*P value*	Inhibition	*P value*
*ABCB1*3435C>T (rs1045642)	CC (76)	234.75±75.84	0.525	0.49±0.26	0.552
	CT (79)	220.80±61.05		0.25±0.17	
	TT (25)	212.32±57.95		0.25±0.13	
*ABCB1*1236C>T (rs1128503)	CC (29)	225.21±77.50	0.879	0.23±0.19	0.667
	CT (92)	227.82±67.99		0.45±0.21	
	TT (59)	222.07±65.61		0.26±0.17	

**Fig 1:**
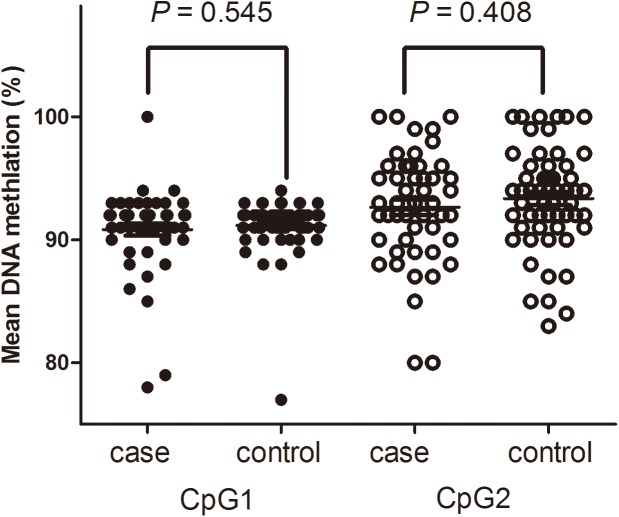
Comparison of platelet function value and the recessive hereditary mode in rs1128503. (1) PRU(CC vs. CT+TT: 225.21±77.50 vs. 226.40±65.77; *P* = 0.689). (2) Inhibition (CC vs. CT+TT(%): 0.23±0.19 vs. 0.34±0.17; P = 0.021).

Because we tested the relationship between platelet activity and the four SNP modes, it became clear that a significant association existed. For ABCB1236C>T (rs1128503), compared with the genotype of CC, the inhibition of platelets in patients with CT+TT was much higher (P = 0.021), which contributed to clopidogrel resistance (***Table 9*** and Fig 1).

**Table 9:** Comparison of platelet function value and the recessive hereditary mode in rs1128503.

	CC (29)	CT+TT (151)	*P value*
PRU	225.21±77.50	226.40±65.77	0.689
Inhibition	0.23±0.19	0.34±0.17	0.021

### The relationship between clopidogrel resistance and methylation levels

**Fig 2:**
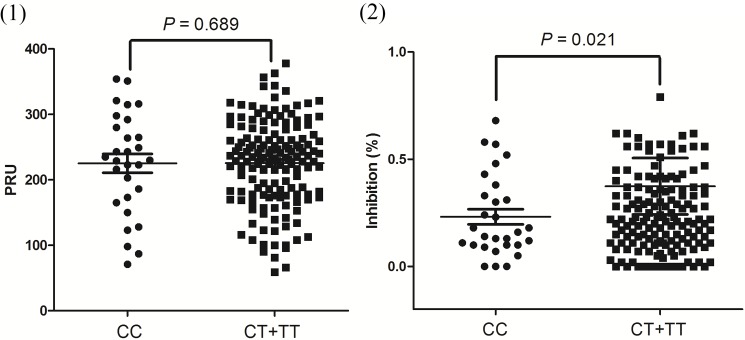
Comparison of ABCB1 methylation levels between cases and controls. ABCB1 CpG1 (cases vs. controls (%):90.84±3.42 vs. 91.18±2.27, *P* = 0.545) and ABCB1 CpG2 (cases vs.controls (%):92.65±4.52 vs. 93.53±4.12, *P* = 0.408).

In the present study, we chose 106 patients at random and found 49 with a poor response to clopidogrel after the platelet function test. We selected a fragment (GRCh37.p13: 87344039–87341039) that contained 2 CpG dinucleotides. Through the bisulphite pyrosequencing assay, we investigated the association of clopidogrel resistance and ABCB1 gene promoter DNA methylation levels in these 106 CAD patients. Additionally, as shown in Fig 2 and ***Table 10***, the methylation levels of CpG1 in ABCB1 in selected fragments were not significantly related to clopidogrel resistance (cases versus controls(%): 90.84±3.42 versus 91.18±2.27, *P* = 0.545) along with ABCB1 CpG2 (cases versus controls (%): 92.65±4.52 versus93.53±4.12, *P* = 0.408). Although we tried to perform a breakdown analysis according to clinical characteristics, we could not find any relationship between the methylation levels of the ABCB1 gene promoter (including CpG1 and CpG2) and poor response to clopidogrel.

**Table 10:** Comparison of ABCB1 methylation levels between cases and controls.

	Cases (49)	Controls (57)	*P value*
CpG1	90.84±3.42	91.18±2.27	0.545
CpG2	92.65±4.52	93.53±4.12	0.408

### Multivariate logistic regression

Respecting the influence of confounding factors, we implemented logistic regression analysis with clinical and genetic variables. The results showed that the TT genotype (rs1045642) was a protective factor of clopidogrel resistance in the homozygotes mode (TT vs. CC:OR = 0.073, P = 0.024). Additionally, the indexes (such as male sex, hyperlipidaemia, HDL, hsCRP, and EF) were inversely correlated with CR, whereas the values of LDL, UA, and the number of stents were associated with CR (***Table 11***).

**Table 11:** The multiple logistic regression analysis of various and homozygote in rs1045642.

	B	Std. Error	Wald	*P value*	Exp(B)	Exp(B) 95% CI
Constant	18.019	17.480	1.063	0.303		
TT vs. CC	-2.621	1.160	5.130	0.024	0.073	0.01–0.71
Gender (male)	-4.072	1.415	8.275	0.004	0.017	0.00–0.27
Hypertension	-0.267	0.955	0.078	0.780	0.766	0.12–4.98
Diabetes mellitus	-1.509	1.490	1.025	0.311	0.221	0.01–4.10
Hyperlipidemia	-3.449	1.142	9.111	0.003	0.032	0.00–0.30
Current smoking	-0.524	1.000	0.274	0.600	0.592	0.08–4.21
Alcohol abuse	2.010	1.106	3.307	0.069	7.466	0.86–65.18
Age	0.086	0.046	3.591	0.058	1.090	1.00–1.19
BMI	-0.060	0.150	0.161	0.688	0.942	0.70–1.26
TC	0.003	0.016	0.046	0.830	1.003	0.97–1.04
TG	0.432	0.359	1.447	0.229	1.540	0.76–3.11
HDL	-6.232	2.466	6.384	0.012	0.002	0.00–0.25
LDL	1.421	0.601	5.583	0.018	4.140	1.27–13.454
GLU	0.092	0.238	0.150	0.698	1.097	0.69–1.75
HbA1c	-0.293	0.166	3.111	0.078	0.746	0.54–1.03
ALT	0.012	0.029	0.176	0.675	1.012	0.96–1.07
AST	0.000	0.004	0.008	0.930	1.000	0.99–1.01
TBIL	-0.033	0.065	0.259	0.611	0.968	0.85–1.10
A	-0.158	0.132	1.434	0.231	0.854	0.66–1.11
BUN	-0.045	0.223	0.041	0.840	0.956	0.62–1.48
CREA	-0.016	0.018	0.838	0.360	0.984	0.95–1.02
UA	0.017	0.006	8.639	0.003	1.017	1.01–1.03
hsCRP	-0.067	0.026	6.839	0.009	0.935	0.89–0.98
PLT	-0.037	0.021	3.134	0.077	0.963	0.92–1.00
MPV	-0.367	0.713	0.264	0.607	0.693	0.17–2.80
PCT	55.804	28.903	3.728	0.054	1.719	0.43–6.88
PDW	-0.190	0.798	0.057	0.812	0.827	0.17–3.96
LEVF	-0.156	0.061	6.516	0.011	0.856	0.76–0.97
Stent	1.592	0.634	6.306	0.012	4.915	1.42–17.03

### Clinical events and genotype in rs1045642 and rs1128503

For these 180 patients, we performed clinical follow-ups for one year. In this year, there were 30 non-fatal myocardial infarctions, 7 non-fatal strokes, 7 cardiovascular deaths, 2 deaths from other causes (1 acute pancreatitis and 1 renal insufficiency), 29 cases of stent thrombosis, and 10 cases of minor or major bleeding. We explored the association between the genotype of rs1045642 and rs1128503 and the risk of cardiovascular events after receiving clopidogrel for one year. The results indicated that the SNP of rs1045642 was related to all-cause mortality (P = 0.024) ***(Table 12***). However, we could not identify the susceptible genotype due to the limited sample size.

**Table 12:** Comparison of clinical events and genotypes in rs1045642 and rs1128503.

	ABCB1 3435C>T (rs1045642)				ABCB1 1236C>T (rs1128503)			
	CC(76)	CT(79)	TT(25)	*P value*	CC(29)	CT(92)	TT(59)	*P value*
MACE	20	16	6	0.669	8	24	12	0.661
No fatal MI	13	13	4	0.990	4	18	8	0.566
No fatal stroke	2	3	2	0.483	1	3	3	0.845
Cardiovascular Death	5	0	2	0.055	3	3	1	0.129
All-cause mortality	7	0	2	0.024	0	1	1	0.342
Stent thrombosis	12	14	3	0.791	5	13	11	0.750
Bleeding	1	7	2	0.101	1	5	4	0.812

## Discussion

The ABCB1gene, located at 7, p21–21.1,[26], encodes the intestinal efflux transporter P-glycoprotein. The P-glycoprotein plays an important role in the bioavailability of multiple endogenous and xenobiotic compounds (such as clopidogrel) from the intestinal lumen via duodenal enterocytes[17]. Recent research revealed that C3435T SNP (rs1045642) was related to an increase in ulcerative colitis, whereas it was associated with tacrolimus pharmacokinetics and the response to platinum-based chemotherapy in lung cancer[27]. Simon *et al*.[18] first analysed the influence of the ABCB1 C3435T polymorphism on clinical outcomes in persons on clopidogrel and found that homozygous patients had an increasing risk of cardiovascular events. However, many other studies showed controversial findings in several subsequent periods. It was reported that the genotype of ABCB1 C1236T (rs1128503) might influence the incidence of breast cancer[28], osteonecrosis[29], and multiple myeloma[30], and the methylation level of the partial ABCB1 gene promoter is closely linked to the efficacy of interventional embolism chemotherapy for cervical cancer[31]. However, there is little information regarding the influence of thers1128503polymorphism and the DNA methylation of selected CpG islands in the ABCB1 gene on clopidogrel’s response.

In the present study, with the application of the Verify-Now P2Y12 assay for platelet function tests in clinical practice and the use of polymerase chain reaction-high performance capillary electrophoresis (PCR-HPCE) for sequencing, we found that if patients had no hypoproteinaemia or had hypertension, the SNP in rs1045642 was associated with CR (CC vs. TT: albumin ≥35, P = 0.042; hypertension, P = 0.045; C vs. T: albumin ≥35, P = 0.033; hypertension, P = 0.040). Additionally, the SNP in rs1045642 was related to the all-cause mortality after one-year follow-up. These data were not consistent with previous research[32] and our most recent meta-analysis[33]. These inconsistent results might be due to the variance of gene frequency in different populations in different areas. Searching the HAPMAP, the information showed that the frequency of the C allele in rs1045642 in Chinese patients was slightly higher than that of Caucasians. A recent study from east asians was indicated that CYP2C19 PM along with ABCB1 3435 TT status was a strong independent predictor of the primary end point (deaths, non-fatal MIs and strokes)[34]. Hence, the variance of the cardinal number in patients with three genotypes resulted in the inconsistent findings compared to the former study. Moreover, to some extent, the limited sample size might have led to statistical bias.

Our results showed that the platelet inhibition of the CT+TT genotype in rs1128503 was larger than that of the CC genotype (P = 0.021).This study was the first to include rs1128503 in the association with the degree of platelet inhibition evaluated by Verify-Now P2Y12 assay. The most recent findings may elicit a novel target and help us to screen the susceptible locus with a new perspective. There are more than 50 single-nucleotide polymorphisms (SNP) in ABCB1. Among them, rs1045642 and rs1128503 are the most common in Chinese patients. Here, we selected these two loci, but we were unable to perform an interaction analysis due to the limited sample size. One study showed that coexisting polymorphisms of the P2Y12 and CYP2C19 genes might be related to persistent platelet activation while on clopidogrel[35]. Another recent study reported that the coexistence of QT mutations/polymorphisms in patients with tetralogy of Fallot (TOF) might aggravate abnormal repolarization after cardiac repair and increase the risks of life-threatening events[36]. Therefore, gene-gene interactions, such as those of CYP3A4 or CYP2C19 or PON1 with ABCB1, need to be studied. Additional studies with larger sample sizes might give us a chance to improve and perfect our limitations.

Applying bisulphite pyrosequencing, we evaluated the DNA methylation levels of two CpG dinucleotides on the ABCB1 promoter region. However, no significance was detected between antiplatelet response and methylation status. Various trials have focused on epigenetics, such as miRNA, siRNA, and DNA methylation. DNA methylation is a reliable epigenetic marker and specifically occurs in the context of cytosine-phosphate guanine (CpG) dinucleotide[37]. The hypermethylation of vertebrate CpG islands (CGIs) is relative to the transcriptional silencing of gene expression and thus controls the protein level[38]. It was found that aberrant methylation plays a vital role in the occurrence and development of diseases, including adrenocortical cancer[39], age-associated cancer[40], coronary artery disease[41], and psychotic disorders[42]. Two years ago, we investigated the contribution of P2Y12 promoter DNA methylation to the risk of clopidogrel resistance (CR) and discovered that the lower methylation of two CpGs indicated that the CR in alcohol abuse and CpG1methylation was inversely correlated to CR in smokers and in the albumin subgroup[24]. To the best of our knowledge, this was the first study on the topic of DNA methylation and CR. The ABCB1 results were not significant, which was inconsistent with another study that showed that hypomethylation of the ABCB1 promoter is related to a poor response to clopidogrel in ischaemic stroke patients[43]. Although there were differences in the study subjects (coronary artery disease and stroke), the population, drug, and the dose were all uniform. These inconsistent findings might be due to the varying regions of CpGIs each study selected. Bisulphite pyrosequencing chose a random region of CpGIs and could not cover the entire area. Thus, sequencing in various regions resulted in inconsistent conclusions. We aim to perform additional studies and a more advanced empirical approach to validate our findings in the future.

The genotype accounts for approximately 2% to 12% of inter-individual variability of the response to clopidogrel[44], and various extrinsic factors (environment, comorbidities, and drug interactions) may also contribute to clopidogrel resistance[45]. We performed logistic regression analysis with confounding variables and showed that male sex and higher levels of albumin and hsCRP decreased the risk of CR, and the number of stents may be positively correlated with CR. These were similar with the study by Gremmel etal, which reported that lower platelet reactivity assessed by the VerifyNow P2Y12 assay were associated with increased levels of hsCRP[46], and the study by Tan etal, which indicated that the number of stents was associated with higher risks of thromboembolic events[47]. Additionally, a recent study showed that chronic kidney disease (CKD) seemed to be associated with poor response to clopidogrel and the high incidence of stent thrombosis in diabetic patients after PCI[48], which might be due to the increased platelet turnover and the up-regulation of the P2Y12 pathway[49]. Hochholzer et al revealed that theCYP2C19*2 polymorphism along with clinical factors (age, diabetes mellitus, body mass index, platelets, verapamil/diltiazem) could replace the phenotyping of platelet function[50]. All these factors demonstrated that the phenomenon of CR was affected by multiple extrinsic and intrinsic factors.

Dual-antiplatelet functions and statins are essential to the proper treatment of CAD, especially after PCI. In follow-up studies, the efficiency of antiplatelet and the influence of statins on atherosclerosis and ischaemic endpoints should be evaluated. If the LDL-C level was greater than 1.3 mmol per litre, the absolute CV risk would decrease in combination with a decrease in LDL-C [51]. A study on IMPROVE-IT reported a clear benefit in the reduction in major cardiovascular (CV) events with simvastatin and ezetimibe therapy[52]. The expression of the gene could also influence the lipid-lowering efficacy, and our most recent meta-analysis indicated that the ABCB1 C3435T polymorphism might be a pharmacogenomic biomarker for predicting clinical outcomes in patients with statins[53]. Therefore, we should consider the effect of statins in the present study, although we discovered that the ABCB1 C3435T polymorphism was related to the all-cause mortality under treatment with clopidogrel in CAD patients. However, the various types of statins and small sample size in our study limited the exploration of the underlying relationship.

Numerous tests are available for measuring platelet function and predicting clopidogrel response, such as the flow cytometric vasodilator-stimulated phosphoprotein phosphorylation (VASP) analysis, the Verify-Now P2Y12 assay, PFA-100, whole blood thromboelastography and impedance aggregometry (multiplate analyzer)[45]. However, various studies in different areas applied distinct methods and cut-off values. Therefore, a common operation standard and evaluation system were urgently required. We produced a systemic and predictive clinical score, which consisted of extrinsic and intrinsic elements, to screen the CR patients. Recently, Fontana[54] and Geisler[55] provided two novel scoring standards, but both of them require further validation. Moreover, if clopidogrel resistance is present, action would be taken to overcome the high platelet reactivity: increasing the loading dose or/and maintenance dose or adding another antiplatelet drug (cilostazol), or changing clopidogrel to a newer thienopyridine, such as prasugrel or ticagrelor. Despite the decline in ischaemic cardiovascular events, these agents produce a higher risk of bleeding [56].

This study presents inherent limitations. First, the sample size was small. Further study with larger sample sizes could be designed for further evaluation. Second, we chose only one fragment of the CGI from the promoter of the ABCB1 gene, and there might be other region correlated to CR. Thus, the conclusions should be interpreted with caution. Third, in gene-gene or gene-drug or gene-environment interactions, unknown confounding factors might exist and affect the expression of the ABCB1 gene, which led to biased results. The exact interactions remain to be investigated in future studies.

## 5.Conclusions

In summary, our study found that the SNP in rs1045642 was associated with CR in patients with hypertension or albumin ≥35 and was related to all-cause mortality. The platelet inhibition of the CT+TT genotype in rs1128503 was larger than that of the CC genotype. Additionally, the methylation of the ABCB1 gene promoter did not affect clopidogrel’s response. Finally, multivariate logistic regression showed that male sex and higher levels of albumin and hsCRP were associated with a decreased risk of CR, and the number of stents may be positively correlated with CR. All these data might provide new insight to elaborate the pathogenesis of CR. However, we aim to perform larger studies with more effective planning and a more advanced empirical approach to validate our findings in further research.
